# Prognostic role of the primary tumour site in patients with operable small intestine and gastrointestinal stromal tumours: a large population-based analysis

**DOI:** 10.18632/oncotarget.23692

**Published:** 2017-12-22

**Authors:** Hua Ye, Hua Xin, Qi Zheng, Qijun Shen, Wenyu Dai, Feng Wu, Cheng Zheng, Ping Chen

**Affiliations:** ^1^ Department of Gastrointestinal and Hernia Ward, Ningbo No. 2 Hospital, Ningbo, Zhejiang, China; ^2^ Clinical Laboratory, Ningbo No. 2 Hospital, Ningbo, Zhejiang, China; ^3^ Department of Health Statistics, Medical School of Ningbo University, Ningbo, Zhejiang, China

**Keywords:** gastrointestinal stromal tumour, GIST, anatomic site, survival, SEER database

## Abstract

The postoperative recurrence risk of gastrointestinal stromal tumour (GIST) should be estimated when considering adjuvant systemic therapy. Previous studies in the literature have suggested that small intestinal GISTs are more aggressive than gastric GISTs. We assessed the prognostic role of the primary tumour site in patients with operable GIST to compare the outcomes of gastric and small intestinal GISTs over a decade of treatment. The Surveillance, Epidemiology, and End Results (SEER) database was queried for cases of gastric and small intestinal GISTs between 2004 and 2014 using the GIST-specific histology code (ICD-O-3 code 8936), and only patients with tissues sampled by surgical resection were selected for this study. Cancer-specific survival (CSS) and overall survival (OS) were compared between small intestinal and gastric GISTs using Cox regression analyses. GISTs were located in the stomach (*n* = 2594, 65%), duodenum (*n* = 228, 6%), and jejunum/ileum (*n* = 1176, 29%). The OS and CSS of patients with GISTs in the duodenum and jejunum/ileum were similar to those of patients with gastric GISTs in Cox regression analyses, except for the CSS of patients with tumour sizes 2.1-5 cm in diameter and ≤ 5 mitoses per 50 HPFs (HR 1.657; 95% CI 1.062-2.587, *p* = 0.026). Tumours sizes 2.1–5 cm in diameter and > 5 mitoses per 50 HPFs (HR 4.627; 95% CI 1.035-20.67, *p* = 0.045) in jejunal/ileal GIST locations had significantly worse CSS than did those in gastric GIST locations. In this large nationwide study, the primary tumour site was not an independent prognostic factor in patients with operable small intestinal and gastric GISTs.

## INTRODUCTION

Gastrointestinal stromal tumours (GISTs) present as the most frequent mesenchymal tumours of the digestive tract. Neoplastic GIST cells originate from usual precursor cells, which are also origins of interstitial cells of Cajal in normal myenteric plexus [1]. The majority of GISTs are primarily located in stomach (60%) and small intestine (30%), followed by duodenum (5%) and colorectum (< 5%). GISTs arising from rectum or colon are rare. Only a small proportion of GISTs (< 1%) occur in oesophagus or appendix. Additionally, rare occasions of extra-gastrointestinal GISTs are generally located in retroperitoneum, omentum or mesentery [2]. Radical resection accompanied by postoperative radiologic follow-up for relapse is the standard regimen for primary, resectable, localised GISTs. Nevertheless, due to the recurrence occurred in a number of patients after radical surgery, administration of imatinib in postoperative stage has been studying to examine its role in decreasing relapse [3]. Imatinib mesylate, a tyrosine kinase inhibitor (TKI), gained approval from the Food and Drug Administration (FDA) for GISTs therapy in 2002 after clinical trials demonstrating that its postoperative use in intermediate- to high-risk subjects prolonged overall survival (OS) and recurrence-free survival (RFS) [4].

Historically, the decision concerning adjuvant treatment has been based on primary tumour size, site, as well as mitotic index. According to common dogma, compared with gastric GISTs, intestinal GISTs are associated with worse prognosis [2, 5]. In the above risk stratification, compared with gastric lesions, intestinal diseases lead to relatively elevated risks of metastasis and tumour-associated mortality. The above-described outcomes present with enormous indications concerning adjuvant therapy in resected GIST patients.

Recently, studies using Surveillance, Epidemiology, and End Results (SEER) database have showed that, unlike these aforementioned previous reports, patients with intestinal GISTs have similar OS as well as cancer-specific survival (CSS) rates with those with gastric GISTs [6, 7]. Given the noted change in GIST incidence, we aimed to investigate the potential effect of site difference on patient outcomes, and determine whether the primary tumour site was a prognostic indicator. Therefore, we extracted data from the SEER database to investigate the outcomes of operable gastric and small intestinal GIST patients after risk adjustment by Cox regression analyses.

## RESULTS

### Baseline characteristics

The cut-off date for follow-up was November 2016, with the median follow-up period of 43 months (ranging from 1 to 131 months). In this study, 3,998 eligible subjects were included for analysis between 2004 and 2014. GISTs were located in stomach in 2594 cases (65%), in duodenum in 228 subjects (6%), and in jejunum/ileum in 1176 cases (29%) (Table 1). For clinicopathological comparisons, the GISTs were categorized into eight subgroups based on maximal tumour diameter and mitotic index per 50 high-power fields (HPFs) as follows: 1) tumours of 2 cm or less in diameter with 5 or fewer mitoses per 50 HPFs; 2) tumours over 2 cm but no more than 5 cm, with 5 or fewer mitoses per 50 HPFs; 3) tumours over 5 cm but no more than 10 cm, with 5 or fewer mitoses per 50 HPFs; 4) tumours over 10 cm, with 5 or fewer mitoses per 50 HPFs; 5) tumours of 2 cm or less, with over 5 mitoses per 50 HPFs; 6) tumours over 2 cm but no more than 5 cm, with over 5 mitoses per 50 HPFs; 7) tumours over 5 cm but no more than 10 cm, with over 5 mitoses per 50 HPFs; and 8) tumours over 10 cm, with over 5 mitoses per 50 HPFs (Table 2).

**Table 1:** The characteristics of 3,998 patients with operable small intestinal and gastric GIST

Characteristic	Gastric GIST (*n* = 2594)	Duodenum GIST (*n* = 228)	jejunum/ileumGIST (*n* = 1176)	statist	*P*
Age, mean ±SD	63.7 ± 13.7	59.1 ± 12.6	60.9 ± 14.3	F = 24.387	0.000
Age				χ^2^ = 38.466	0.000
≤ 60	1012 (39.0%)	125 (54.8%)	557 (47.4%)		
> 60	1582 (61.0%)	103 (45.2%)	619 (52.6%)		
Race				χ^2^ = 162.556	0.000
White	1654 (63.8%)	175 (76.8%)	954 (81.1%)		
Black	571 (22.0%)	12 (5.3%)	92 (7.8%)		
Other^*^	350 (13.5%)	39 (17.1%)	125 (10.6%)		
Unknown	19 (0.7%)	2 (0.8%)	5 (0.5%)		
Sex				χ^2^ = 9.110	0.011
Male	1293 (49.8%)	125 (54.8%)	645 (54.8%)		
Femal	1301 (50.2%)	103 (45.2%)	531 (45.2%)		
TumorSize				χ^2^ = 120.878	0.000
≤ 2 cm	300 (11.6%)	20 (8.7%)	76 (6.5%)		
2.1–5 cm	897 (34.6%)	97 (42.5%)	267 (22.7%)		
5.1–10 cm	824 (31.8%)	81 (35.5%)	470 (40.0%)		
> 10 cm	573 (22.0%)	30 (13.2%)	363 (30.8%)		
Mitiotic Count				χ^2^ = 5.457	0.65
≤ 5 per 50HPF	2276 (87.7%)	202 (88.6%)	1001 (85.1%)		
> 5 per 50HPF	318 (12.3%)	26 (11.4%)	175 (14.9%)		

NOTE:^*^American Indian/AK Native, Asian/Pacific Islander. Categoric variables were compared by using the Chi-square test, and continuous variables were compared by using One-way ANOVA test. Abbreviation: SD, standard deviation.

**Table 2:** Baseline characteristics of patients With GIST size and mitotic count type

	≤ 5 per 50HPF				> 5 per 50HPF							
Characteristic	≤ 2 cm (*n* = 386)	2.1–5 cm (*n* = 1149)	5.1–10 cm (*n* = 1176)	> 10 cm (*n* = 768)	χ^2^	*p*	≤ 2 cm (*n* = 10)	2.1–5 cm (*n* = 112)	5.1–10 cm (*n* = 199)	> 10 cm (*n* = 198)	χ2	*p*
Age					11.647	0.009					4.930	0.177
≤ 60	170 (44.0%)	446 (38.8%)	516 (43.9%)	354 (46.1%)			5 (50.0%)	36 (32.1%)	79 (66.4%)	88 (44.4%)		
> 60	216 (56.0%)	703 (61.2%)	660 (56.1%)	414 (53.9%)			5 (50.0%)	76 (67.9%)	120 (60.3%)	110 (55.6%)		
Race					20.724	0.014					8.867^a^	0.473
White	291 (75.4%)	828 (72.1%)	794 (67.5%)	513 (66.8%)			5 (50.0%)	73 (65.2%)	143 (71.9%)	136 (68.7%)		
Black	52 (13.5%)	178 (15.5%)	195 (16.6%)	152 (19.8%)			3 (30.0%)	23 (20.5%)	31 (15.6%)	41 (20.7%)		
Other^*^	40 (10.4%)	137 (11.9%)	176 (15.0%)	99 (12.9%)			2 (20.0%)	15 (13.4%)	25 (12.5%)	20 (10.1%)		
Unknown	3 (0.7%)	6 (0.5%)	11 (0.9%)	4 (0.5%)			0 (0%)	1 (0.9%)	0 (0%)	1 (0.5%)		
Location					86.238	0.000					40.132	0.000
Gastric	291 (75.4%)	807 (70.2%)	711 (60.5%)	467 (60.8%)			9 (90.0%)	90 (80.4%)	113 (56.8%)	106 (53.5%)		
Duodenum	19 (4.9%)	89 (7.7%)	69 (5.9%)	25 (3.3%)			1 (10.0%)	8 (7.1%)	12 (6.0%)	5 (2.5%)		
jejunum/ileum	76 (19.7%)	253 (22.1%)	396 (33.6%)	276 (35.9%)			0 (0%)	14 (12.5%)	74 (37.2%)	87 (44.0%)		

NOTE:^*^American Indian/AK Native, Asian/Pacific Islander, Categoric variables were compared by using the Chi-square test and Fisher exact test.

a: Fisher exact test.

### Effect of GIST location on survival in the SEER database

**Figure 1:**
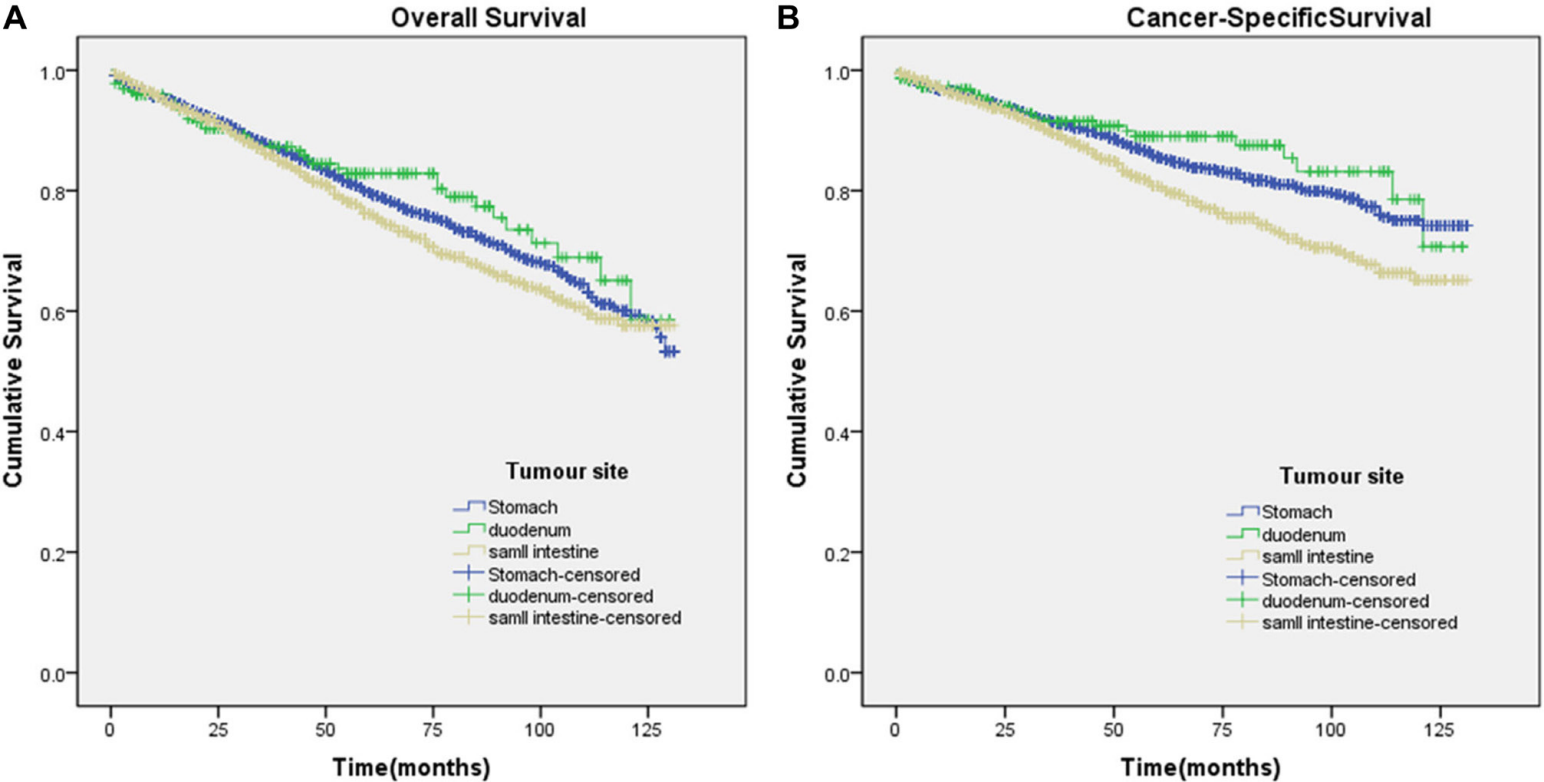
Kaplan-Meier curves for overall and cancer-specific survival Panel (**A** and **B**) depict the overall and cancer-specific survival in the original data set and panel. (A) χ^2^ = 4.303 (*P* = 0.116); (B) χ^2^ = 16.08 (*P* < 0.001).

In univariate analyses, compared with gastric and duodenal GISTs, jejunal/ileal GISTs were significantly associated with worse CSS (Figure 1A, 1B). In univariate Cox regression analyses, there were no significant differences in OS (hazard ratio (HR) 1.143; 95% confidence interval (CI) 0.983–328, *p* = 0.081) between jejunal/ileal and gastric GISTs, but there were significant differences in CSS (HR 1.388; 95% CI 1.162–1.656, *p* = 0.000) between jejunal/ileal and gastric GISTs. Conversely, there were no significant differences in OS (HR 1.087; 95% CI 0.788–1.501, *p* = 0.611) or in CSS (HR 0.988; 95% CI 0.649–1.503, *p* = 0.954) between duodenal and gastric GISTs (Table 3).

**Table 3:** Univariate Cox regression survival analysis for evaluating the influence of GIST location on survival in SEER database

Primary site	OS		CSS	
	HR (95% CI)	*p*	HR (95% CI)	*p*
stomach	1 (reference)		1 (reference)	
duodenum	0.877 (0.638–1.207)	0.422	0.810 (0.535–1.228)	0.321
jejunum/ileum	1.143 (0.983–1.328)	0.81	1.388 (1.162–1.656)	0.000

### Subgroup analysis for evaluating the effects of GIST location according to mitotic index and size

**Figure 2:**
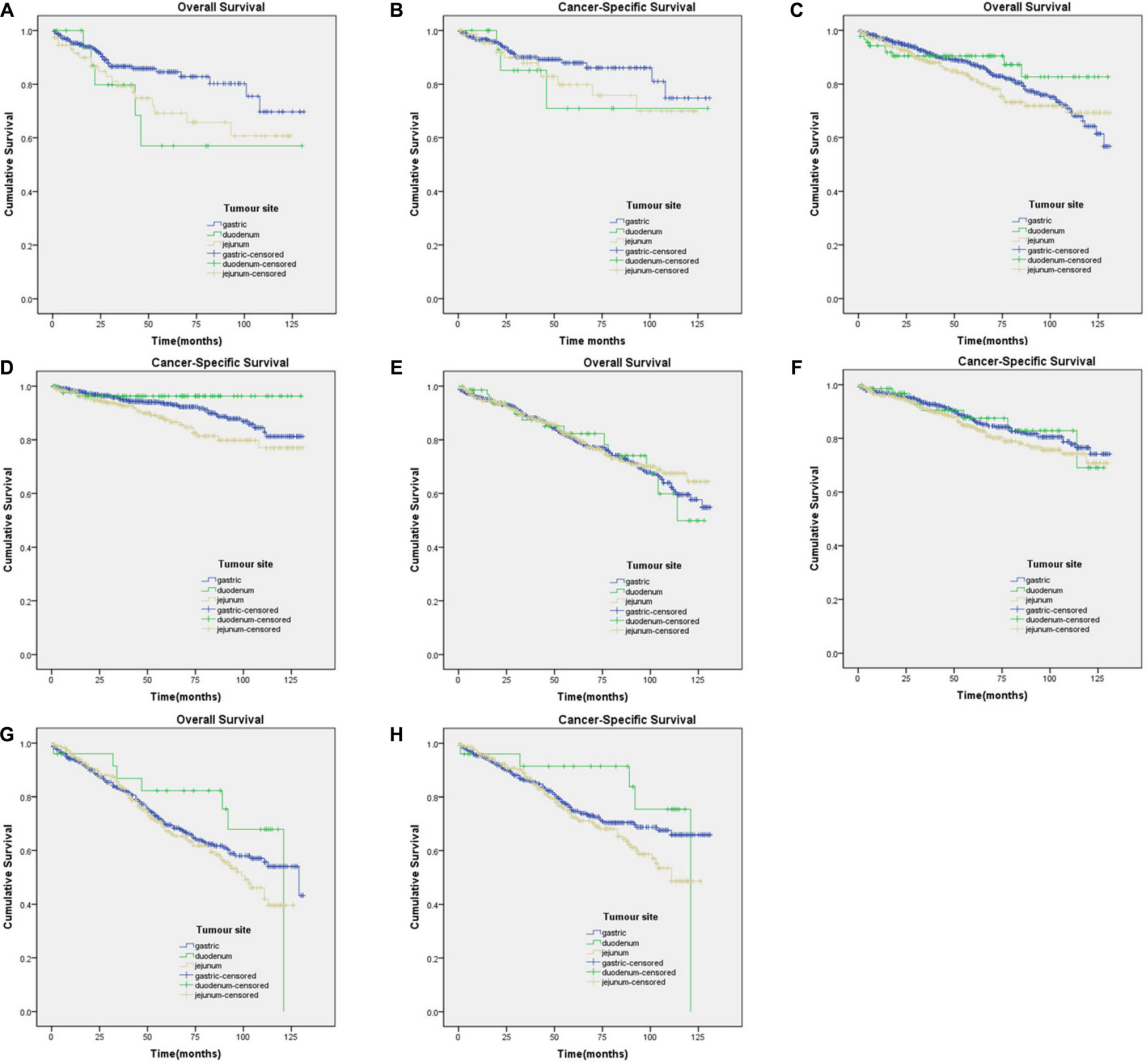
Kaplan-Meier curves for overall and cancer-specific survival in patients with ≤ 5 mitoses per 50 HPF to subgroup1)-4) (**A**) OS in subgroup1) χ^2^ = 5.774 (*P* = 0.056); (**B**) CSS in subgroup1) χ^2^ = 1.799 (*P* = 0.407); (**C**) OS in subgroup2) χ^2^ = 2.500 (*P* = 0.287); (**D**) CSS in subgroup2) χ^2^ = 8.259 (*P* = 0.016); (**E**) OS in subgroup3) χ^2^ = 0.286 (*P* = 0.867); (**F**) CSS in subgroup3) χ^2^ = 1.990 (*P* = 0.370); (**G**) OS in subgroup4) χ^2^ = 2.813 (*P* = 0.245); (**H**) CSS in subgroup4) χ^2^ = 3.691 (*P* = 0.158).

**Figure 3:**
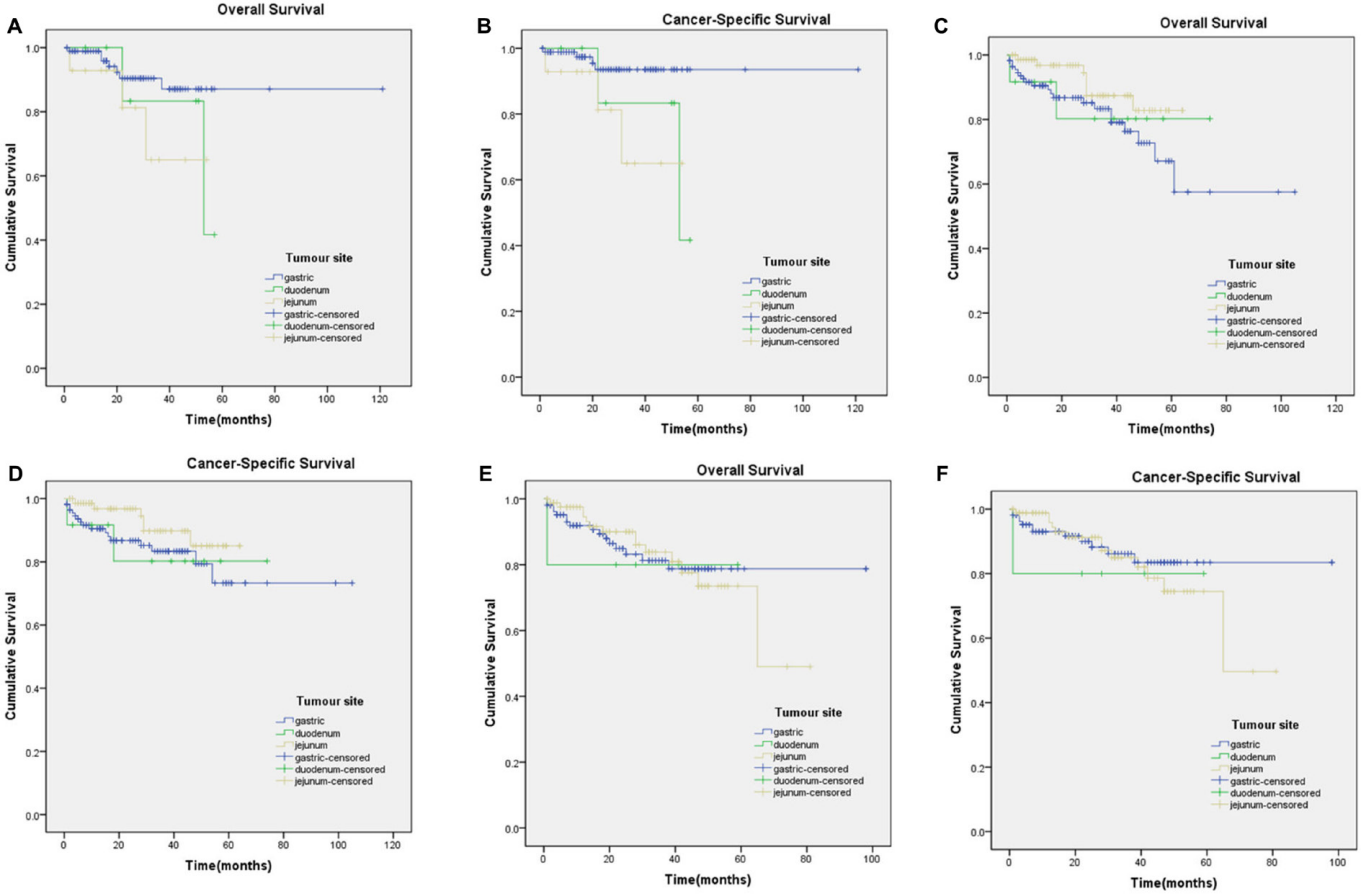
Kaplan-Meier curves for overall and cancer-specific survival in patients with > 5 mitoses per 50 HPFs to subgroup6)-8) (**A**) OS in subgroup6) χ^2^ = 2.784 (*P* = 0.249); (**B**) CSS in subgroup 6) χ^2^ = 5.661 (*P* = 0.059); (**C**) OS in subgroup7) χ^2^ = 3.108 (*P* = 0.211); (**D**) CSS in subgroup7) χ^2^ = 2.384 (*P* = 0.304); (**E**) OS in subgroup8) χ^2^ = 0.048 (*P* = 0.976); (**F**) CSS in subgroup8) χ^2^ = 0.397 (*P* = 0.820).

The Kaplan-Meier curves for subgroups were displayed in Figure 2A–2H, Figure 3A–3F. Subgroup 2) GISTs were significantly associated with worse CSS (Figure 2D), whereas survival was not significantly different in GISTs patients in the other subgroups. Subgroup 5), which contained tumours of 2 cm or smaller with over 5 mitoses per 50 HPFs, had insufficient data for the study.

The effects of GIST location on survival were further analysed on patients with equal mitotic index and tumour size, using the Cox proportional hazards regression model to exclude any feasible confounding factors (Table 4). Patients with jejunal/ileal GISTs had a significantly worse CSS than those with gastric GISTs in only two subgroups, namely Subgroup 2) (HR 1.657; 95% CI 1.062–2.587, *p* = 0.026) and Subgroup 6) (HR 4.627; 95% CI 1.035–20.67, *p* = 0.045). In contrast, there were no apparent differences in OS and CSS between GIST locations of stomach, duodenum and jejunum/ileum in any other subgroups. Generally, gastric GISTs had a more favourable prognosis than intestinal GISTs harbouring similar parameters. However, after the analysis was adjusted for the mitotic index and tumour size variables, the primary tumour site was not an independent prognostic indicator in subjects with operable small intestinal and gastric GISTs.

**Table 4:** Cox proportional hazards model for overall survival and cancer–specific survival in patients with operable gist by mitotic index, size, and site

variable	≤ 5 per 50HPF				> 5 per 50HPF			
	OS		CSS		OS		CSS	
	HR (95% CI)	*p*	HR (95% CI)	*p*	HR (95% CI)	*p*	HR (95% CI)	*p*
≤ 2 cm					Insufficient data		Insufficient data	NI
stomach	1 (reference)		1 (reference)		-		-	
duodenum	2.121 (0.831–5.414)	0.116	1.714 (0.519–5.663)	0.377	-		-	
jejunum/ileum	1.798 (1.025–3.154))	0.041	1.490 (0.748–2.967)	0.256	-		-	
2.1–5cm								
stomach	1 (reference)		1 (reference)		1 (reference)		1 (reference)	
duodenum	0729 (0.381–1.396)	0.341	0.447 (0.140–1.433)	0.175	2.209 (0.452–10.805)	0.328	3.830 (0.686–21.392)	0.126
jejunum/ileum	1.210 (0.855–1.712)	0.282	1.657 (1.062–2.587)	0.026	2.776 (0.715–10.774)	0.140	4.627 (1.035–20.677)	0.045
5.1–10 cm								
stomach	1 (reference)		1 (reference)		1 (reference)		1 (reference)	
duodenum	1.021 (0.591–1.766)	0.940	0.993 (0.481–2.046)	0.984	0.759 (0.178–3.242)	0.710	0.979 (0.226–4.245)	0.978
jejunum/ileum	0.933 (0.712–1.221)	0.612	1.253 (0.909–1.728)	0.168	0.472 (0.200–1.113)	0.086	0.492 (0.194–1.248)	0.135
> 10 cm								
stomach	1 (reference)		1 (reference)		1 (reference)		1 (reference)	
duodenum	0.671 (0.313–1.435)	0.303	0.651 (0.265–1.599)	0.349	1.234 (0.163–9.318)	0.839	1.671 (0.217–12.878)	0.622
jejunum/ileum	1.165 (0.897–1.512)	0.251	1.256 (0.934–1.689)	1.256	0.984 (0.480–2.018)	0.965	1.211 (0.552–2.657)	0.634

NI: not included in Cox regression analysis.

## DISCUSSION

The present population-based study, which included 3,998 eligible patients, supplied convincing evidence that duodenal GIST patients harboured outcomes similar to gastric GIST subjects. Further analyses revealed that patients in only two subgroups of intestinal GISTs had worse prognosis than those with gastric GISTs; while there were no significant differences between gastric GISTs and intestinal GISTs in any of the other subgroups. Thus, we suggest that the primary tumour site is not an independent prognostic indicator in patients with operable intestinal, duodenal and gastric GISTs, which is opposed to present guidelines as well as common beliefs. Therefore, it is mitotic index and tumour size rather than tumour location that should be taken into primary consideration in deciding for or against adjuvant therapy of imatinib in duodenal and intestinal GISTs.

A comprehensive understanding of prognostic factors in operable primary GISTs might be beneficial for proper risk classification, which is of great help in determination of postoperative follow-up approaches as well as the necessity of adjuvant treatment. Tumour size and mitotic index exert as the most essential and universally applied prognostic indicators in GISTs, which were the foundations of a consensus approach to risk classification of GISTs, as published in 2002 [8]. One of the tenets of the therapeutic approaches, which is based on the belief of malignant potential in all GISTs, is facilitated by three large-scale retrospective researches by Miettinen et al. at the Armed Forces Institute of Pathology (AFIP) [9–11]. Together, these studies comprised of the largest published series of GISTs categorized by current criteria, from which long-term clinical follow-up data were accessible for the pre-imatinib era. The 2002 consensus criteria for risk classification of GISTs were verified and expanded by the above-described studies. In 2006, Miettinen and Lasota published the generally adopted AFIP risk classification, indicating that the risk classification of primary GISTs should be based upon tumour size, mitotic index as well as tumour location [2]. Dematteo et al. recently demonstrated that mitotic index, tumour size, as well as tumour site could be to independently predict recurrence of primary GIST patients without TKI therapy, who previously received radical surgery (small intestinal GIST subjects have the greatest risk) [12]. The AFIP risk stratification was even incorporated into ESMO guidelines in 2012 [13]. According to the AFIP risk stratification, intestinal GIST patients harbour significantly worse prognosis than gastric GIST subjects, especially when the tumour size is over 5 cm. Moreover, patients with duodenal and rectal GISTs have worse outcomes. Gold from the Memorial Sloan-Kettering Cancer Center (MSKCC) described a nomogram using tumour size, tumour site, as well as mitotic rate in predicting RFS after exsection of localised primary GISTs[14], which were well calibrated. However, the discriminatory capacity of the nomogram was not increased after including tyrosine kinase mutation status, which might be due to the number of subjects enrolled in its development.

However, a recent study has showed that unlike the aforementioned previous reports, patients with intestinal GISTs have similar OS and CSS rates to those with gastric GISTs [6, 7]. In spite of the identification of tumour size and mitotic index as prognostic indicators by several large-scale studies of surgically radical GISTs, there is still controversy over the prognostic significance of anatomic site in GISTs. In this study, we highlighted the importance of this finding given that a commonly used prognostic classification system, the AFIP classification, assigns a higher risk of tumour metastases or tumour-related death to duodenal, especially jejunal/ileal GISTs [2, 6]. In our current study, we found that only two subgroups, namely, tumour size 2.1–5 cm in diameter and ≤ 5 mitoses per 50 HPFs (HR 1.657; 95% CI 1.062–2.587, *p* = 0.026) and those with > 5 mitoses per 50 HPFs (HR 4.627; 95% CI 1.035–20.67, *p* = 0.045), in jejunal/ileal GIST locations had significantly worse CSS than did those in gastric GIST locations. There is great difference between our findings and the outcomes of AFIP risk classification, and some results differ from the paper by Guller et al. It is unknown why our findings are different from the data by Miettinen, et al., who definitely performed prominent pioneering work over the understanding of the pathophysiology and therapy of GISTs, as well as Guller's data from the SEER database. The different time periods might be responsible for the difference, during which, different subjects were enrolled. Specifically, the AFIP risk stratification included subjects through 1970 until 1996, while patients from 1998 until 2011were enrolled in Guller's research. However, our analyses only assessed patients in the SEER database between 2004 and 2014, which reflected the time period after imatinib was approved by FDA for use in GIST treatment. We analysed the effects of GIST location on survival in curatively resected patients with equal mitotic indexes and tumour sizes using the Cox proportional hazards regression model to eliminate underlying confounding factors; additionally, the exclusion criteria were different from those in the aforementioned study [2, 6, 7]. Regardless, it is definitely defective to only rely on a risk stratification with continuous biological variables, including mitotic index and tumour. Therefore, it is of great help to utilize prognostic contour maps, as proposed by Joensuu [15], to assess recurrence risk in GIST patients. Prognostic contour or heat maps might be more appropriate to estimate individual outcome, which is likely to exhibit wide application than than AFIP risk table as well as the MSKCC nomogram. Additionally, these maps take tumour site into account.

The limitations of the present study should be acknowledged. One limitation of the present study is the lack of information regarding RFS in the SEER database. Secondly, data on TKIs used and pathologic outcomes, such as tumour necrosis, ulceration, type of PDGFR or KIT mutations, are not available in the SEER database. Thirdly, the applied models are simplified with accessible and acceptable measurements, which definitely do not include adequate variables involved in outcomes of patients. Finally, despite of the risk adjustment for known confounders, potential bias due to unknown confounding factors cannot be excluded.

In conclusion, in contrast to common belief, the primary tumour site is not an independent prognostic indicator in subjects with operable small intestinal and gastric GISTs, which harbours related indications in determination of adjuvant therapy in small intestinal GIST subjects. Due to the population-based nature of this analysis, which mirrors the actual US population with GISTs, the absence of certain types of information does not impact our results, however, it does limit the extent to which our data can be interpreted.

## MATERIALS AND METHODS

### Origins of materials

The SEER registry, sponsored by the National Cancer Institute, collects information on cancer incidence and survival. The current SEER database (from 2004–2014) consists of 18 population-based cancer registries that represent approximately 28% of the United States population. The SEER data contain no identifiers and are publicly available for studies of cancer-based epidemiology and health policy. We obtained permission to access the research data (Reference Number: 10266-Nov 2016).

### Inclusion and exclusion criteria

The specific inclusion criteria were as follows: the year of diagnosis ranged from 2004 to 2014; GIST was defined by GI tumour site codes and the GIST-specific histology code (ICD-O-3 code 8936); and only patients with tissues sampled by surgical resection. The exclusion criteria were as follows: patients with cancer diagnosis at autopsy or on the death certificate only; patients without histological confirmation; patients without documentation of age at diagnosis or patients younger than 18 years; patients with other SEER-reportable cancers unless the GIST was the first diagnosed malignancy so that the analyses of CSS were more accessible; patients who survived less than 1 month (such patients may die from surgical complications or they may experience rapid progression after palliative resection); GIST sizes coded and mitotic counts without records; and patients with oesophageal (*N* = 19) GISTs (the patient numbers were too small for good statistics).

### Statistical analysis

The age at diagnosis, gender, race, site record, histological type, survival in months, tumour size, mitotic count, and cause of death were retrieved from the SEER database. The outcomes of interest in this study included OS and CSS, which were determined according to specific codes provided by SEER. Death attributed to other causes was defined as a censored observation.

Comparisons among groups for categorical variables were analysed by Pearson's χ^2^ test or Fisher's exact test, and those for continuous variables were analysed by using one-way ANOVA test, as appropriate. Estimates of survival rates were generated using the Kaplan-Meier method, and the differences in survival rates were evaluated with log-rank tests. Adjusted HRs along with 95% CIs were calculated by using the Cox proportional hazards model. All statistical analyses were two-sided, and *p* < 0.05 was indicative of statistical significance. All statistical analyses were performed with SPSS software (Statistical Package for the Social Sciences, IBM SPSS Statistics, version 22 for Macintosh; IBM, Armonk, NY).
